# How do fertility intentions lead to contraceptive continuation among a cohort of family planning users who received services from the private sector in Nigeria

**DOI:** 10.12688/gatesopenres.13253.2

**Published:** 2022-05-06

**Authors:** Sara Chace Dwyer, Aparna Jain, Sikiru Baruwa, Emeka Okafor, Daini Babajide Oluseyi, Osimhen Ubuane

**Affiliations:** 1 Population Council, Washington DC, USA; 2 Population Council, Abuja, Nigeria; 3 Society for Family Health, Abuja, Nigeria

**Keywords:** Family planning, fertility intentions, drug shops, pharmacies, task sharing, private sector, Nigeria

## Abstract

**Background: **The Federal Ministry of Health of Nigeria is exploring task sharing family planning (FP) services to Community Pharmacists (CPs) and Patent and Proprietary Medicine Vendors (PPMVs). Yet few studies have explored contraceptive continuation of clients who received FP services from pharmacies and drug shops. This paper uses longitudinal data and looks at women’s contraceptive continuation approximately nine months after they received FP services from CPs and PPMVs in Kaduna and Lagos states.

**Methods:** Longitudinal data for this analysis come from an evaluation of the IntegratE project. 491 women were interviewed within 10 days after receiving a FP service from an IntegratE CP or PPMV and approximately nine months later. The dependent variable is contraceptive continuation at the follow-up interview and the independent variable is fertility intentions as reported at enrollment. Multivariate logistic regression models were used to assess the association between fertility intentions and contraceptive continuation.

**Results: **89% of women continued using contraception approximately 9 months after the enrollment interview. Women who intended to have a child in more than two years were significantly more likely to continue using contraception compared to women who intended to have a child within two-years (AOR 2.6; 95% CI 1.1-6.1). Among women who were asked about the quality of care received, 93% said the CP/PPMV asked whether they wanted to have a/another child in the future and 85% said they were asked when they would like to have that child.

**Conclusion: **The fertility intentions of women who seek FP services from CPs and PPMVs in Nigeria can predict contraceptive continuation. As Nigeria task shares FP services to CPs and PPMVs, training on comprehensive FP counseling will be essential for scale-up. Since many women continued using FP, CPs and PPMVs have the potential to expand access to, and support women’s continued use of, FP.

## Introduction

Contraception allows women and couples to plan for their families by spacing or limiting births. Avoiding unintended pregnancies also contributes to improvements in maternal and newborn health outcomes ^1^. In Nigeria, about 15% of all women of reproductive age (15–49) have an unmet need for family planning (FP)- 10% for spacing and 5% for limiting pregnancies ^2^. Yet as of 2018, 14% of women were using any method and 11% were using a modern contraceptive ^2^. In addition to the barriers facing women in initiating contraception, discontinuation has been found to contribute to approximately one-third of unmet need globally ^3^. About 41% of Nigerian women who begin using a contraceptive method discontinue that method within 12-months ^2^. The main reasons Nigerian women reported discontinuing their method include desire to become pregnant, side effects/health concerns and infrequent sex ^2^.

Previous studies have documented factors associated with contractive discontinuation while in need (i.e. wanting to delay or prevent pregnancy). Method-related factors include experience of side effects ^4– 8^, the type of method selected (for example, short-acting versus long-acting or hormonal versus nonhormonal methods) ^5, 9, 10^, and women’s satisfaction with the method ^11^. Experience of side effects is the most commonly cited reason for method discontinuation while in need ^4– 8, 11^, although some studies have found that the effect of side effects on discontinuation may be overestimated ^9, 12, 13^.

Individuals’ characteristics, intimate relationships, and social factors can also influence whether a woman continues to use her method. Non-method related factors associated with contraceptive continuation include: a previous unintended pregnancy, partner support, age, number of desired children, number of living children, number of male children, being in school or working, and discussing FP with a friend ^8, 9, 11, 13^. Studies have also shown that a woman’s motivation to prevent pregnancy and intent to use contraception are associated with method selection ^14^ and contraceptive continuation ^9, 15^.

Fertility intentions, that is the desire for a certain number of children and the intended timing of a first birth and subsequent spacing between births ^16^, are also associated with contraceptive continuation. A longitudinal study in Indonesia found that women who wanted to wait at least two years before their next pregnancy were more likely to continue using a modern contraceptive than those who wanted a birth within two years ^17^. Fertility intentions also play important roles in women's decision to use contraception and can predict birth outcomes ^16, 18^. For example, a study in Senegal demonstrated that women who did not want additional children were less likely to become pregnant, especially if they were also using a modern method ^18^. Previous studies have found that factors including age, parity, perception of a partner's desire for additional children, pregnancy attitudes, and a previous negative birth experience are associated with women’s fertility intentions ^16, 19, 20^. Yet for most women, fertility intentions often fluctuate and can even change within a short period of time ^18, 19, 21^. For example, Jones *et al*. found that while 39% of women who were interviewed three times over the course of a year reported being uncertain if they wanted more children, only 9% consistently reported uncertainty at all three interviews. In longitudinal study in India, among women who reported wanting a child in two or more years during an enrollment interview, only 5% of those women consistently reported that preference during subsequent follow-up interviews within a 12-month period ^21^. Similarly, 14% of those who reported wanting children within two years did so consistently during follow-up interviews ^21^.

Given that fertility intentions can influence contraceptive use and continuation, and often fluctuate, researchers have highlighted the importance of discussing fertility intentions during FP counseling and general healthcare visits ^17, 19, 21– 23^. Quality counseling, in itself, has been shown to improve uptake and continuation of FP. For example, higher levels of quality of care received during FP counseling was significantly associated with contraceptive continuation in a study conducted in Indonesia ^17^ and in India ^22^. Jain *et al*. found that the method selection domain of quality of care, which includes two items related to the provider’s conversation around their client’s fertility intentions, was significantly associated with contraceptive continuation three months later ^22^.

To date, few studies have explored contraceptive continuation of clients who received FP services from private sector pharmacies and drug shops. This paper uses longitudinal data from Nigeria and looks women’s contraceptive continuation approximately nine months after they received FP services from private sector Community Pharmacists (CPs) and Patent and Proprietary Medicine Vendors (PPMVs) in Kaduna and Lagos states. Specifically, this paper aimed to assess the fertility intentions of women seeking services from CPs and PPMVs and whether their fertility intentions lead to continued contraceptive use nine months later.

### Expanding family planning access in Nigeria through the private sector, the IntegratE Project

As part of their strategy to expand access to FP in Nigeria, the Federal Ministry of Health is exploring task sharing certain FP services to CPs and PPMVs. Task sharing involves delegating or distributing specific tasks among healthcare teams and, where appropriate, from high-skilled health care workers to those with fewer qualifications ^24^. Task sharing within public sector is common, especially the provision of oral and injectable contraceptive by community health workers ^25, 26^. Task sharing FP to private sector pharmacies and drug shops, however, has been limited in many countries despite being identified a promising high impact practice to expand access to FP ^27^ and that drug shop owners generally similar education qualifications as community health workers and pharmacists hold higher education degrees ^28^.

In Nigeria, while CPs and PPMVs are not formally recognized as FP service providers, they are important sources for primary health care ^29, 30^ and for FP: 22% of modern contraceptive users’ report receiving their last method from a PPMV and 12% from a private pharmacy ^2^. PPMVs are not required to receive a standard training or a degree for licensure and are currently authorized to provide over the counter medications only ^30, 31^. To become a CP, you most complete a five-year degree in Pharmacy by a university recognized by the Pharmacists Council of Nigeria.

Previous studies have shown that while many PPMVs provide FP services, that they do not have the required knowledge to provide FP services such as oral and injectable contraceptives ^32, 33^. One study found that knowledge to provide injectable contraceptives increased with training however ^34, 35^ and other studies have found clients are generally satisfied with the FP services received from these providers ^32, 34^.

In collaboration with the Federal Ministry of Health, IntegratE, a four-year project (2017–2021), is piloting a three-tiered accreditation system. This system seeks to stratify PPMVs in accordance with their prior health training. Those without health qualifications are categorized as Tier one, those with qualifications in Nursing and Midwifery, Community Health Extension and Community Health Officers are categorized as Tier two and those with pharmacy technician’s certificate are categorized as Tier three. Under the pilot accreditation system, Tier one PPMVs participated in a three-day training on FP counseling, provision of condoms, cycle beads and oral contraceptive refills, referrals for all other FP methods, and documenting FP services. Tier two and Tier three PPMVs received the same training as Tier one PPMVs plus an additional three-day training on injectable administration, and implant insertion and removal. As CPs already participate in a formal pharmacy program, they function outside of the pilot accreditation system but received the same training as Tier two and Tier three PPMVs.

Between July 2018 and September 2019, 894 CPs and PPMVs enrolled in the project and were trained in FP based on their tier. All trainings were classroom-based and then CPs, Tier two and Tier three PPMVs also participated in clinical sessions at nearby public health facilities. During the clinical sessions, they were required to competently complete 12 implant insertions and three removals before providing these services at their businesses. The IntegratE project and state teams (Pharmacy Council of Nigeria, National Association of Patent and Proprietary Medicine Dealers, State Ministry of Health and State Primary Health Care Development Agency) provided supportive supervision approximately three months after the training.

### Study sites

As of 2020, the IntegratE project is implemented in Lagos and Kaduna. Lagos state, with a population of 9,013,534, is in the southern part of Nigeria while Kaduna state, with population of 6,113,503, is in northern part of the country ^36^. According to the 2018 Nigeria Demographic and Health Survey, the total fertility rate is relatively low in Lagos state (3.4 births per woman) compared to Kaduna state (5.9 births per woman). In terms of fertility intentions, 23% of women interviewed in Lagos state reported desiring no additional children, 10% were unsure, and 66% wanted additional children (18% within two years, 14% in more than two years, and 34% were unsure of the timing) ^2^. In Kaduna, 18% of women reported desiring no additional children, 80% wanted additional children (37% within two years, 23% in more than two years, and 20% were unsure of the timing) ^2^. Less than 1% of women in Kaduna were unsure whether or not they wanted additional children ^2^. The modern contraceptive prevalence rate among married women of reproductive age varies across the two states from 29% in Lagos to 14% percent in Kaduna ^2^. About 33% of women in Lagos and 34% in Kaduna who begin using a contraceptive method discontinue that method within 12-months ^2^.

## Methods

### Data source

Longitudinal data used for this analysis come from an on-going evaluation of the IntegratE project (2018–2021). The IntegratE project is funded by the Bill & Melinda Gates Foundation and MSD for Mothers. As part of this evaluation, women who received FP services (counseling, referral, condoms, and/or oral, injectable and implant contraceptives) from an IntegratE-trained CP or PPMV were interviewed over the phone within 10 days after receiving the service and approximately nine months later. Women were interviewed within 10 days of their visit to reduce recall bias related to women’s experiences with the services received from the CP and PPMV. Verbal informed consent was received before the beginning enrollment and follow-up interview. Written consent was not obtained as interviews were conducted over the phone. The research protocol, including informed consent procedures, received ethical approval from the Population Council’s Institutional Review Board (Protocol 878) and Nigeria’s National Health Research Ethic Committee.

From June to November 2019, IntegratE CPs and PPMVs in Kaduna and Lagos states provided female FP clients between the ages of 16–49 with basic information about the study and requested their permission to collect and share their contact details with the interviewers part of the research team. All interviewers participated in a training on research ethics, the study’s design and objectives, procedures for implementing informed consent forms, and conducting telephone interviews. Interviewers called clients who agreed to share their information with the research team to confirm their eligibility. Eligible respondents were between 16–49 (women 16–17 had to be married to be considered an emancipated minor), received a FP service from a trained CP or PPMV, and owned their own cell phone. Respondents could be continuing FP users, method switchers, or new to FP altogether. If eligible, trained data collectors provided potential respondents with details of the study including the objectives, what was expected of them as a respondent, and potential risks and benefits to participating. Those who agreed were interviewed over the phone and compensated 500 Naira (Approximately 1.39 USD) in phone credit. Interviewers also requested to re-contact participants.

Interviewers used tablets to administer a quantitative client enrollment interview that included questions on socio-demographic characteristics, current and previous contraceptive use, quality of care received, and general perceptions on their experiences receiving services from the CP or PPMV. The same respondents were interviewed by phone approximately 8–11 months after that initial interview and were asked questions related to their current contraceptive use, experience of side effects, continued use of FP services from CPs and PPMVs, and COVID-19. A total of 596 women were interviewed at an enrollment and 517 at 9–11 months later, (13% lost to follow-up).

### Dependent variable

The dependent variable is contraceptive continuation at the follow-up interview. Respondents who received a method or referral from a IntegratE CP or PPMV were asked if they were currently using the same, different, or no method to avoid or delay pregnancy. We dichotomized the dependent variable where respondents who reported using the same method and or those who switched to a different FP method are considered FP continuers and coded as one. This includes methods they purchased directly from the CP or PPMV or received because of a referral. Respondents who stopped using FP altogether are considered discontinuers and coded as 0.

### Independent variable

The main independent variable is fertility intentions as reported at enrollment. A categorical variable was created from two questions: “ *do you want to have any (more) children*” and those who said yes were then asked, “ *when would you like to have your first/next child*.” The independent variable is coded as follows: 1= respondents who reported they want a child within the 2 years of the interview; 2= respondents who reported they want a child two years or more from the interview; 3= respondents who want a child but do not know when; and 4= respondents who do not want any/more children.

Additional covariates explored and included in the multivariate model include age, education, marital status, number of living children, employment status, past contraceptive use, experience of side effects at follow-up and state. Ten observations were missing from the question “ *what is the highest level of education that you have attained*.” For the education variable, missing observations included in the category for did not achieve a secondary education or higher/did not respond. For experience of side effects, we created a categorical variable. At follow-up, contraceptive users of the pill, injectable or implant were asked if they were currently experiencing side effects, and if they responded No, they were then asked if they had experience side effects since the enrollment interview. Those who responded Yes to currently experiencing side effects or experience of side effects since enrollment were coded as 1 and those who said No were coded as 0. Those who did not respond or were skipped from this question because they were using another method (for example, cycle beads or condoms) were coded as 2 No Response. All women who discontinued their method were also asked whether they experience side effects as a result of their method. Those who said Yes were coded as 1, those who said No were coded as 0 and anyone who did not responded were coded as 2.

### Data analysis

The analytical sample was limited to respondents who were interviewed at enrollment and follow-up, were 16–49 years of age, and either who began to use or continued to use a FP method as a result of their visit to a CP or PPMV (n=491). Respondents who were pregnant at the time of the follow-up interview (n=17), those who were 50 of older (n=5), those who did not receive a method from a CP/PPMV or their referral (n=3), and who did not answer the fertility intentions questions (n=1) were excluded. Descriptive statistics were calculated for respondent characteristics, FP use, fertility intentions at enrollment, and experience of side effects as reported at follow-up. Multivariate models that accounted for the longitudinal nature of the data were conducted but the likelihood ratio test showed that most of variance in the random intercept was accounted for by the covariates. Multivariate logistic regression models were used to assess the association between fertility intentions and contraceptive continuation. Descriptive statistics were also calculated for two quality of care indicators related to fertility intentions among those who were asked about the quality of care received.

We conducted three sensitivity analyses. First, we conducted Pearson chi2 tests to compare respondent characteristics of all women interviewed at enrollment versus those included in the analytical sample and there were no significant differences between the two samples in the characteristics reported. We then ran the multivariate logistic regression model including pregnant women in the sample, and then with women age 16–53. The results were similar to the final model in both sensitivity analyses. The analyses were conducted in STATA.SE, Version 16.

## Results

Table 1 presents the demographic profile of women who received FP services from CPs and PPMVs at the enrollment interview. Just under half of the women were 25–34 years of age (47%). Many (75%) had attained at least a secondary education and 72% were employed at the time of the enrollment interview. Most women were married (95%) and had two or more living children (86%). A little over half (56%) had previously used FP in the past and expressed a desire for a/additional children (56%): 18% wanted a child within the next two years, 35% wanted a child in two or more years and 4% were unsure of the timing. Half of the women were from Kaduna and half from Lagos. At follow-up, one-third (33%) reported experiencing side effects and approximately 89% of women continued to use contraception (82% reported using the same method and 7% reported switching to another method, data not shown).

**Table 1.** Respondent characteristics at enrollment, and experience of side effects and contraceptive continuation and at follow-up (n=491).

	%
**Age**	
16–24	13.9
25–34	46.8
35–49	39.3
**Education**	
Did not complete a secondary school education/no response	25.3
Completed secondary education or higher degree	75.7
**Currently employed**	
Yes	72.3
No	27.7
**Marital status**	
Never married/single	4.3
Married/in-union	94.7
Separated/widowed	1.0
**Number of living children**	
None	3.5
1	10.6
2	18.7
3	28.1
4+	39.1
**Has used FP in the past**	
Yes	55.6
No	44.4
**Fertility intentions**	
Wants a child within 2 years	17.5
Wants a child in 2 or more years	34.6
Wants a child, don’t know when	3.7
Does not want (more) children	44.2
**Experienced of side effects as reported at follow-up**	
Yes	33.0
No	62.7
Did not say	4.3
**Continued using contraception at follow-up**	
Yes	89.4
No	10.6
**State**	
Kaduna	50.1
Lagos	49.9

**Figure 1.**
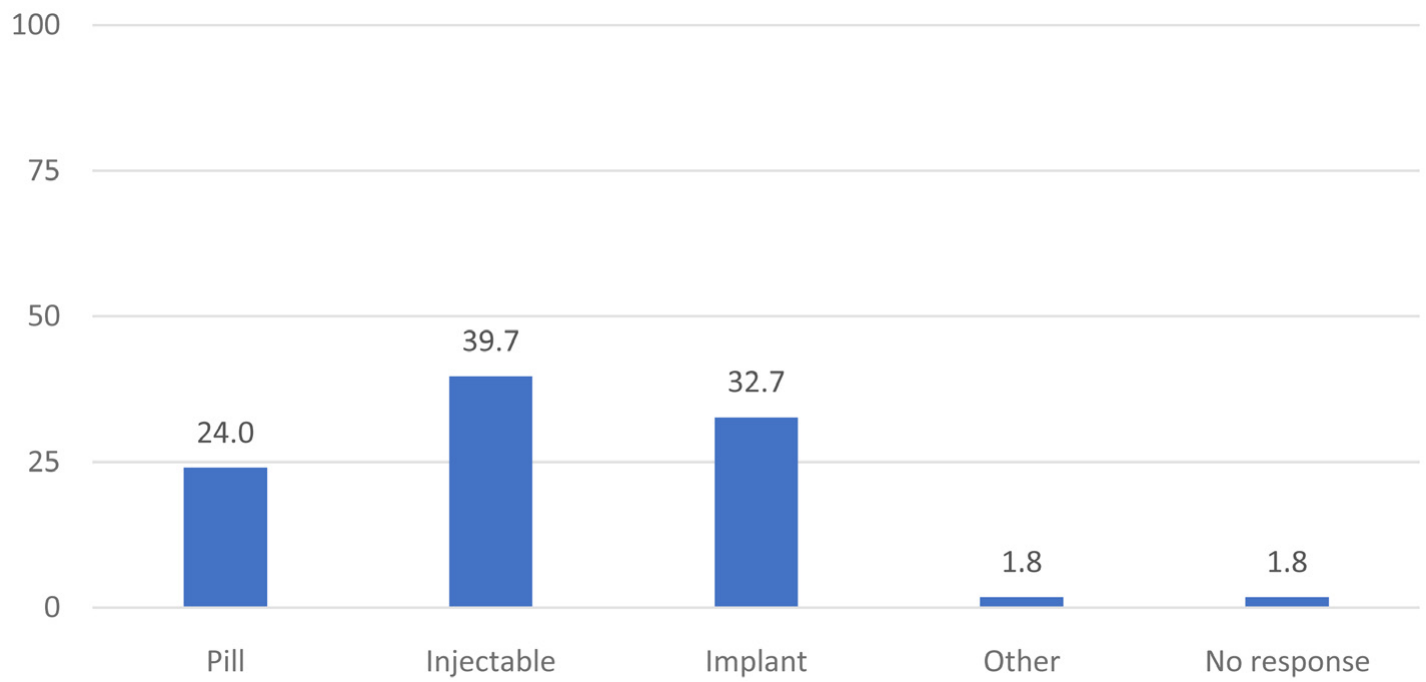
Distribution of current method use as reported at enrollment (N=491).

Figure 1 shows the distribution of current method use as reported at the enrollment interview. Two-fifths (40%) of women were using the injectable, 33% were using an implant and 24% were using the pill. Two percent reported using another method such as condoms, cycle beads, and or an IUD (as a results of a referral).

**Figure 2.**
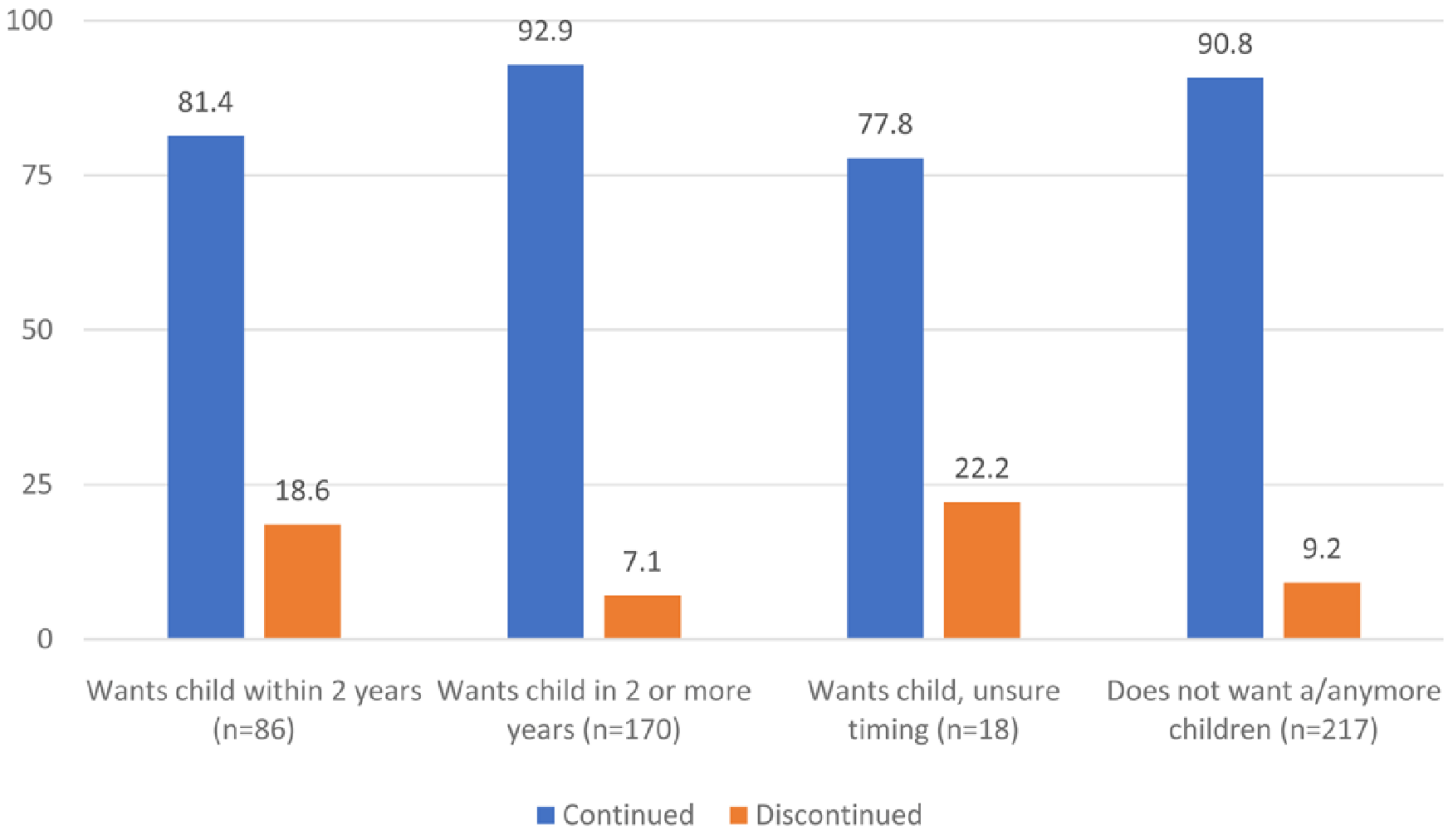
Distribution of continuers and discontinuers at follow-up by their fertility intentions as reported at enrollment (N=491).

Figure 2 shows the distribution of continuers and discontinuers at follow-up by their fertility intentions as reported at enrollment. Among women who wanted to have a child within two years, 81% continued using contraception approximately nine months later compared to 93% of women who reported wanting a child in more than two years and 91% of women who did not want any more children. About 78% of women who were unsure when they wanted their next child continued using their contraceptive method approximately nine months later.

Unadjusted and adjusted odds ratios of contraceptive continuation are presented in Table 2. Women who wanted their next child more than two years after enrollment were three times more likely to continue using contraception compared to those who wanted a child within two years (OR=3.0; 95% CI 1.4-6.7). Women who did not want any/more children were two times more likely to continue using contraception compared to those who wanted a child within two years (OR=2.3; 95% CI 1.1-4.6). When accounting for covariates in the multivariate model, women who intended to have a child in more than two years remained significantly more likely to continue using contraception compared to women who intended to have a child within two-years, although the odds ratio decreased slightly (AOR 2.6; 95% CI 1.1-6.1). Not wanting any/more children was no longer significant in the multivariate model but the OR remained in the expected direction.

**Table 2.** Univariate and multivariate logistic regression models of fertility intentions on contraceptive continuation among women who received family planning services from Community Pharmacists and Patent and Proprietary Medicine Vendors (n=491).

	Univariate Model		Multivariate Model	
	OR	95% CI	OR	95% CI
**Fertility intentions**				
Want a child within 2 years from now	ref	-	ref	-
Want a child 2 or more years from now	3.00 **	1.35-6.70	3.00 **	1.35-6.70
Want a/another child but don’t know when	0.64	0.20-2.03	0.64	0.20-2.03
Do not want a/ additional children	2.25 *	1.11-4.59	2.25 *	1.11-4.59
**Age**				
16–24	ref	-	ref	-
25–34	0.95	0.39-2.29	0.95	0.39-2.29
35–49	0.94	0.38-2.32	0.94	0.38-2.32
**Education**				
Did not complete a secondary school education/no response	ref	-	ref	-
Completed secondary education or higher degree	0.59	0.28-1.25	0.59	0.28-1.25
**Currently employed**				
Yes	1.04	0.55-1.95	1.04	0.55-1.95
No	ref	-	ref	-
**Marital status**				
Never married/single	ref	-	ref	-
Married/in-union	1.42	0.40-5.00	1.42	0.40-5.00
Separated/widowed	0.67	0.54-8.20	0.67	0.54-8.20
**Number of living children**				
None	0.20 **	0.06-0.65	0.20 **	0.06-0.65
1	0.56	0.21-1.45	0.56	0.21-1.45
2	0.56	0.25-1.26	0.56	0.25-1.26
3	0.75	0.35-1.61	0.75	0.35-1.61
4+	ref	-	ref	-
**Have ever used family planning**				
Yes	1.14	0.64-2.01	1.14	0.64-2.01
No	ref	-	ref	-
**Experience of side effects**				
Yes	ref	-	ref	-
No	2.14 *	1.15-3.96	2.14 *	1.15-3.96
Did not respond	0.27 **	0.10-0.71	0.27 **	0.10-0.71
**State**				
Lagos	ref	-	ref	-
Kaduna	1.59	0.89-2.83	1.59	0.89-2.83

* p-value ≥ 0.05; ** p-value ≥ 0.01

Women who had no children were 80% less likely to be using contraception compared to women with 4 or more child (OR 0.20; 95% CI 0.06-0.65) and this association remained significant in the multivariate model (AOR 0.16; 95% CI 0.03-0.97). Women who reported that they did not experience side effects at follow-up were two times more likely to be using contraception compared to women who did in both the univariate model (OR 2.1; 95% CI 1.2-4.0) and multivariate model (AOR 2.1; 95% CI 1.1-4.0).

## Discussion

Results from this analysis suggest that among women who received services from CPs and PPMVs in Nigeria, fertility intentions are associated with their contraceptive continuation. Specifically, the respondents in this study were more likely to continue using contraception if they reported wanting a child in two or more years compared to women who wanted a child within two years. These results are consistent with previous studies that have found associations between fertility intentions and contraceptive use and continuation ^17, 37, 38^. We also found that experience side effects and number of living children were also associated with contraceptive continuation for the women in this study, consistent with existing literature ^4– 8, 11, 20^.

The contraceptive users in this study were similar in age to contraceptive users broadly in Nigeria, although were slightly more education: 75% of women in this study completed at least a secondary education compared to 60% nationally ^2^. As FP services are task shared to CPs and PPMVs, the results from this study underscore the importance of discussing fertility intentions during FP counseling between CPs/PPMVs and their clients in order to help women choose a method that best suits their needs and to continue using FP. As fertility intentions change over time ^15, 19^, and stronger or more precise fertility intentions have been shown to be associated with the actualization of those intentions ^39^, CPs and PPMVs should also discuss fertility intentions with their clients often, consistent with recommendations from studies in Sweden ^23^, and the U.S. ^19^.

The association between fertility intentions and continuation remained significant even when accounting for experience of side effects. This suggests that understanding a woman’s fertility intentions, in addition to providing information about side effects, is essential for comprehensive FP counseling and quality of care ^17, 22^. Many of the women in this study who were asked about the quality of care received from the CP or PPMV that they saw reported that the CP or PPMV asked about their fertility intentions (93% said they were asked whether they wanted to have a or another child in the future and 85% were asked when they would like to have a or another child, data not shown). This suggests that when CPs and PPMVs are trained in FP counseling, they can provide quality counseling and facilitate a dialogue around fertility intentions with their clients, despite the additional time it may take to discuss fertility intentions with their clients. As the method selection domain of quality of care has also been shown to be significantly associated with continuation ^22^, emphasizing the importance of comprehensive FP counseling when task sharing FP service provision to CPs and PPMVs will be essential. Understanding other aspects in a woman’s life, such as number of living children, will also be important for these new FP providers in aiding their clients in choosing an appropriate method. It is important to note, however, that many women chose CPs and PPMVs for the perceived discretion and privacy ^29, 34^. Ensuring women are comfortable discussing these issues with these private sector outlets, is also an important consideration.

Another important finding from this study is that 89% of women in need who received a FP method as a result of a visit from CPs and PPMVs continued to use their method about nine months later. These results suggest that when CPs and PPMVs are properly trained, they have potential to support their clients who wish to continue using contraception, even as about two-thirds of the women interviewed were using short acting methods such as the pill, injectable, or condom. Previous studies have documented proximity and flexible operating hours as reasons why women prefer PPMVs for FP and other primary health care services ^40, 41^. Therefore, the accessibility of CPs and PPMVs may also facilitate women’s continued use FP.

### Limitations

Data for this analysis came from a specific population. All women interviewed sought FP services from the private sector and therefore the results are not representative of the broader population of Nigerian women of reproductive age. The women in this study may also have been more motivated to use contraception since they sought FP services. Our results, however, are consistent with other studies that have found an association between fertility intentions and contraceptive continuation. The results from this study, therefore, add a unique perspective of women who seek services from private sector pharmacies and drug shops.

Another limitation is that respondents had to own a phone to be eligible to participate, which may have introduced a potential source of selection bias. This criterium was included to reduce the proportion of respondents lost to follow-up since we used phone interviews.

## Conclusion

The fertility intentions of women who seek FP services from private sector pharmacies and drug shops (PPMVs) in Nigeria can predict contraceptive continuation. With training, CPs and PPMVs can discuss fertility intentions with their clients and many women who seek services from these providers continue using FP, even short acting methods like the pill or injectable. As task sharing FP services to these private sector cadres is scaled in Nigeria and in similar settings, supporting CPs and PPMVs to provide comprehensive FP counseling, emphasizing the importance of discussing fertility intentions with women often, will be essential.

## Data availability

### Underlying data

Harvard Dataverse. How do fertility intentions lead to contraceptive continuation among a cohort of family planning users who received services from the private sector in Nigeria. The dataset analyzed during the current study will be available on the Population Council’s site: https://dataverse.harvard.edu/dataverse/popcouncil in January 2022 as data for this paper came from a larger evaluation and data collection is ongoing. They will also be available from the corresponding author on reasonable request.

## Author’s contributions

SCD managed the overall preparation of the manuscript including conceptualizing and drafting the manuscript and conducting the analysis and contributed the development of the overall study’s methodology. SB supervised the implementation of the research activities and contributed to drafting the manuscript. EO led the funding acquisition for the Integrate project, including the study, and reviewed drafts of the manuscript. UO and DBO managed the coordination of data collection and data management. AJ was the principal investigator and led the development of the overall study’s methodology, and also guided the conceptualization of the manuscript and analysis. All authors have read and approved this version of the manuscript.
