# Supplementary material for: How do fertility intentions lead to contraceptive continuation among a cohort of family planning users who received services from the private sector in Nigeria
Source: Gates Open Res. 2022 May 6;5:103. Originally published 2021 Jul 19. [Version 2] doi: 10.12688/gatesopenres.13253.2 (PMC9086212; doi:10.12688/gatesopenres.13253.2)
Supplement: Peer review history

# Peer review: Reviewer response for version 2

**DOI:** 10.21956/gatesopenres.14897.r32036

**Published:** 2022-05-09

**Authors:** Jill Peterson

**Affiliations:** 1 FHI 360, Washington, DC, USA

Thank you for addressing the previous comments. Two final comments from me:

1. There is a typo in the new sentence in the discussion related to age and education - "The contraceptive users in this study were similar in age to contraceptive users broadly in Nigeria, although **were slightly more education**:.." - should be "were slightly more educated", or "had slightly more education". 
2. It would be important to note which results were not statistically significant and therefore removed from the model. You could then say that those variables were then analyzed descriptively. 

Is the work clearly and accurately presented and does it cite the current literature?

Yes

If applicable, is the statistical analysis and its interpretation appropriate?

I cannot comment. A qualified statistician is required.

Are all the source data underlying the results available to ensure full reproducibility?

Yes

Is the study design appropriate and is the work technically sound?

Yes

Are the conclusions drawn adequately supported by the results?

Partly

Are sufficient details of methods and analysis provided to allow replication by others?

Yes

Reviewer Expertise:

Family planning continuation, pharmacy provision of family planning, family planning service denial/turnaway

I confirm that I have read this submission and believe that I have an appropriate level of expertise to confirm that it is of an acceptable scientific standard.

# Peer review: Reviewer response for version 1

**DOI:** 10.21956/gatesopenres.14488.r31323

**Published:** 2021-11-23

**Authors:** Jill Peterson

**Affiliations:** 1 FHI 360, Washington, DC, USA

This is a very interesting and important article looking at the ability of Community Pharmacists and Patent Proprietary Medicine Vendors in two states of Nigeria to provide FP that meets the needs of those seeking it. Specifically, the research examines whether the fertility intentions of women in the sample can predict contraceptive continuation. 

Specific comments: 

- It would be helpful to mention who funds the IntegratE project. 
- A table presenting the information found in the study sites would help guide the reader through all the data. You could then add a bit of information about demographic similarities/differences of the two states, including information about religion, wealth quintile, etc., to help readers who are unfamiliar with Nigeria. As I read further, your sample seems highly educational and with high employment rates.  Does your sample reflect the employment and educational averages for the states, or is this a factor of where they sought their FP? 
- Why no mention of the finding of those who did not report on side effects as being less likely to be using contraception at follow-up? 
- Are you missing an "and" after (Protocol 878)? 
- I'm confused about Figure 3. It seems like it should come higher up in the results. I'm also confused about an n of 411 in Figure 3 (the number asked about their fertility intentions), but when you break down fertility intentions by continuation in figure 2, you have a higher n (n=491). Is the difference being asked fertility intentions by the data collector vs. the provider (CP or PPMV)? If so, that could be made more clear to the reader. Is it being used as a proxy for quality of care? Were other proxy indicators for quality of care collected, such as counseling on side effects?
- I'm also confused as to why having been asked about their fertility intentions by the provider was not included as an independent variable. In the discussion you highlight the importance of discussing fertility intentions during counseling, but this is not a variable I see listed in your analysis. The same is true of providing information about side effects. I don't see this variable in your model, but you mention its importance in the discussion. 

I recommend clarification on the last two points to ensure that the discussion and conclusions flow from the results.

Is the work clearly and accurately presented and does it cite the current literature?

Yes

If applicable, is the statistical analysis and its interpretation appropriate?

I cannot comment. A qualified statistician is required.

Are all the source data underlying the results available to ensure full reproducibility?

Yes

Is the study design appropriate and is the work technically sound?

Yes

Are the conclusions drawn adequately supported by the results?

Partly

Are sufficient details of methods and analysis provided to allow replication by others?

Yes

Reviewer Expertise:

Family planning continuation, pharmacy provision of family planning, family planning service denial/turnaway

I confirm that I have read this submission and believe that I have an appropriate level of expertise to confirm that it is of an acceptable scientific standard, however I have significant reservations, as outlined above.

# Response

**Published:** 2022-04-24

**Authors:** Sara Chace Dwyer

**Affiliations:** Population Council, USA

Specific comments: 

- It would be helpful to mention who funds the IntegratE project. 
  - **Response:** Funding was added on page 6.
- A table presenting the information found in the study sites would help guide the reader through all the data. You could then add a bit of information about demographic similarities/differences of the two states, including information about religion, wealth quintile, etc., to help readers who are unfamiliar with Nigeria. As I read further, your sample seems highly educational and with high employment rates.  Does your sample reflect the employment and educational averages for the states, or is this a factor of where they sought their FP? 
  - **Response:** As the data come from two different sources, we presented the data in the paragraph for simplicity. We added a sentence to in Discussion section to highlight that our sample was similar in age to contraceptive users broadly, while our sample was slightly more educated.
- Why no mention of the finding of those who did not report on side effects as being less likely to be using contraception at follow-up?
  - **Response:** This finding is presented the Results on page 13 and in the Discussion on page 14.
- Are you missing an "and" after (Protocol 878)? 
  - **Response**: We added accordingly.
- I'm confused about Figure 3. It seems like it should come higher up in the results. I'm also confused about an n of 411 in Figure 3 (the number asked about their fertility intentions), but when you break down fertility intentions by continuation in figure 2, you have a higher n (n=491). Is the difference being asked fertility intentions by the data collector vs. the provider (CP or PPMV)? If so, that could be made clearer to the reader. Is it being used as a proxy for quality of care? Were other proxy indicators for quality of care collected, such as counseling on side effects?
  - **Response:** We agree and removed Figure 3 from the results. We did add the proportions to the discussion on page 14 to highlight that providers in this study did ask about women’s fertility intentions, which is promising given the findings of this study.
- I'm also confused as to why having been asked about their fertility intentions by the provider was not included as an independent variable. In the discussion you highlight the importance of discussing fertility intentions during counseling, but this is not a variable I see listed in your analysis. The same is true of providing information about side effects. I don't see this variable in your model, but you mention its importance in the discussion. 
  - **Response:** We included the quality-of-care questions related to provider discussion around fertility intentions and side, but these results were not statistically significant and therefore removed from the model. As other papers have found a link between counseling and continuation, we added these results descriptively demonstrate CPs and PPMVs are a potential access point for counseling and contraceptive use.

# Peer review: Reviewer response for version 1

**DOI:** 10.21956/gatesopenres.14488.r31328

**Published:** 2021-11-22

**Authors:** Julie Hernandez

**Affiliations:** 1 Department of International Health and Sustainable Development, School of Public Health and Tropical Medicine, Tulane University, New Orleans, LA, USA

This manuscript focuses on an important component of family planning strategies by assessing the role of providers, and specifically private sector providers, in method continuation. With discontinuation being a large contributor to unmet need worldwide, identifying individual and environmental factors that could limit its incidence among FP users is crucial to effective contraceptive programming.

 Overall, this paper is of high quality. The study justification is comprehensive, and variables of interest are clearly introduced. The study context is clearly presented and details the relevance of private sector providers (often overlooked in FP service delivery strategies). Data analysis and results are sound and rigorously explained.

 The main issue the reviewer would like to raise is the risk of stretching the results regarding the importance of private sector providers. While it is well-documented that they are preferred sources of contraceptives in many Sub-Saharan African countries, the study design, in the absence of a control group, does not allow to compare continuation rates for women who received their method from a CP/PPMV *vs *another service delivery provider.

 Some sentences in the manuscript should thus be nuanced, e.g.:

- (Results): *"These results suggest that when CPs and PPMVs are properly trained, many of their clients continue to use contraception".* In the absence of a control group, this statement goes beyond what the study design can demonstrate.
- (Limitations): " *The results from this study, therefore, add a unique perspective of women who seek services from private sector pharmacies and drug shops*."

Furthermore, experiments conducted in other countries to strengthen the role of private sector outlets suggested that increased training and role in FP counselling came at several costs: on the SDP’s side, the additional counselling adds to the workload of the providers who may be reluctant to engage in more than business/sales transactions. On the client side, women indeed value private outlets for their “proximity and flexible hours” (as mentioned in the manuscript) but also for their discretion and the capacity to conduct a rapid and inconspicuous business exchange. Being asked additional (intimate) questions about fertility intention might decrease their intention to frequent these outlets.

 A mention of those limitations and a more nuanced consideration of the possible roles of private sector providers, considering existing evidence, would be welcome in the Discussion section of the manuscript.

 The key main point, that fertility intentions are the main determinant of contraceptive use and continuation, remains valid and extremely important to the field.

**Minor improvements:**

- Methods: *– *Since the CPs and PPMVs were in charge of initially recruiting participants, how did you ensure the absence of bias in who the providers approached/ignored to participate in the study (e.g. skewed towards more educated, older, “most likely to continue” participants?).
- This may go beyond the scope of this manuscript but recent research on the role of side-effects in method discontinuation suggests that binary variables (“Did you experience side effects? Yes / No) are insufficient to grasp the influence of these side-effects in method (dis-)continuation. More specific questions on the intensity, length and “bearable” nature of those side-effects are much more effective predictors of future contraceptive behaviors.
* *
- In Data Analysis, clarify *“were using FP because of their visit to a CP or PPMV”, makes it sound too restrictive, earlier in the Methods it sounds like women who initiated FP anywhere but continued resupplying their methods at a participating CP / PPMV could be recruited in the study as well? *

Results:

- In the sample description (socio-demographic characteristics), it would be interesting to comment on study participants’ profile compared to average contraceptive users/women with unmet need in Nigeria (based on DHS or PMA survey)? This would also help addressing the question of possible recruitment bias raised above.
- Figures 1 and 2 might be unnecessary? Results could be presented in a table or simple paragraph.
- Nuance “Only 74% of women who were unsure...” as this group represents only 3.7% of the sample, the validity of the findings must be nuanced.
- Clarify the sentence: *“As stronger or more precise fertility intentions have been shown to be associated with the actualization of those intentions and that fertility intentions changed over time.”*  There seem to be two separate statements and/or a fragment of the sentence is missing?
- Considering that a large share of women (almost a third) used implants, which are more difficult to “discontinue” (they require visit to health facilities and payment off fees at a minimum), the authors could have included sub-analysis of continuation patterns based on method used at baseline.

Is the work clearly and accurately presented and does it cite the current literature?

Yes

If applicable, is the statistical analysis and its interpretation appropriate?

Yes

Are all the source data underlying the results available to ensure full reproducibility?

Yes

Is the study design appropriate and is the work technically sound?

Yes

Are the conclusions drawn adequately supported by the results?

Partly

Are sufficient details of methods and analysis provided to allow replication by others?

Yes

Reviewer Expertise:

International family planning, Democratic Republic of Congo

I confirm that I have read this submission and believe that I have an appropriate level of expertise to confirm that it is of an acceptable scientific standard.

# Response

**Published:** 2022-04-24

**Authors:** Sara Chace Dwyer

**Affiliations:** Population Council, USA

A mention of those limitations and a more nuanced consideration of the possible roles of private sector providers, considering existing evidence, would be welcome in the Discussion section of the manuscript.

  

- **Response: **We thank the reviewer for this feedback and included on page 14 is a sentence that underscores that many women reported being asked about their fertility intentions, suggesting that these providers did take the time for this component of FP counseling. Based on our experience with this, and other projects working with PPMVs in Nigeria specifically, we believe that there is an incentive to provide quality services in order to facilitate policy change, which would in turn also benefit their business and standing in the community. We added a sentence on page 15 to highlight the importance of client privacy.

The key main point, that fertility intentions are the main determinant of contraceptive use and continuation, remains valid and extremely important to the field.

**Minor improvements:**

- Methods: *– *Since the CPs and PPMVs were in charge of initially recruiting participants, how did you ensure the absence of bias in who the providers approached/ignored to participate in the study (e.g. skewed towards more educated, older, “most likely to continue” participants?).
  - **Response:** Meetings were held with CPs and PPMVs enrolled in this study to review the goals of the study, what information to provide to clients, and the importance of asking clients’ permission to share their information with a member of the research team. Informed consent, confirmation of eligibility criteria and enrollment was completed by a member of the research team. The inclusion criteria were also clearly discussed with CPs and PPMVs and we found that some women did not want to participate or did not own a phone independent of another family member.
- This may go beyond the scope of this manuscript but recent research on the role of side-effects in method discontinuation suggests that binary variables (“Did you experience side effects? Yes / No) are insufficient to grasp the influence of these side-effects in method (dis-)continuation. More specific questions on the intensity, length and “bearable” nature of those side-effects are much more effective predictors of future contraceptive behaviors. 
* *
  - **Response:** We thank the reviewer for this thoughtful comment. You raise a good question and we agree with the reviewer on the importance of these nuanced questions related to experience of side effects. We chose, however, not to add these questions due to the intended focus of the evaluation study. Another aspect for consideration is that women enrolled in this study could be continued users or new users (new episode of use or new to contraceptives). As such, women who were continuing with contraceptive may no longer experience side effects or have decided to continue to use even with the experience of side effects.

 

- In Data Analysis, clarify *“were using FP because of their visit to a CP or PPMV”, makes it sound too restrictive, earlier in the Methods it sounds like women who initiated FP anywhere but continued resupplying their methods at a participating CP / PPMV could be recruited in the study as well? *
  - **Response:** That is correct. We changed the language to “ *who began to use or continued to use a FP method as a result of their visit to a CP or PPMV*”

Results:

- In the sample description (socio-demographic characteristics), it would be interesting to comment on study participants’ profile compared to average contraceptive users/women with unmet need in Nigeria (based on DHS or PMA survey)? This would also help addressing the question of possible recruitment bias raised above.
  - **Response:** We added some details related to the age and education of the women in our sample compared to contraceptive users in Nigeria in the second paragraph of the discussion section. It is important to note that this study was conducted among current users, and therefore is not representative of the broader Nigerian population.
- Figures 1 and 2 might be unnecessary? Results could be presented in a table or simple paragraph.
  - **Response:** Thank for this feedback. We added the figures to add some graphical representation in the article.
- Nuance “Only 74% of women who were unsure...” as this group represents only 3.7% of the sample, the validity of the findings must be nuanced.
  - **Response:** We updated sentence by removing the word “only” and simply stated the finding. 
** **
- Clarify the sentence: *“As stronger or more precise fertility intentions have been shown to be associated with the actualization of those intentions and that fertility intentions changed over time.”*  There seem to be two separate statements and/or a fragment of the sentence is missing?
  - **Response:** We updated the sentence to read “ *As fertility intentions change over time and stronger or more precise fertility intentions have been shown to be associated with the actualization of those intentions, CPs and PPMVs should also discuss fertility intentions with their clients often, consistent with recommendations from studies in Sweden and the U.S.”*

 

- Considering that a large share of women (almost a third) used implants, which are more difficult to “discontinue” (they require visit to health facilities and payment off fees at a minimum), the authors could have included sub-analysis of continuation patterns based on method used at baseline.
  - **Response:** We looked at method discontinuation and found no statistical difference by method adopted. We highlight on page 5 that CPs and PPMVs were also trained in implant removal.
